# Comparison of Solar Radiation and Myopia Occurrence in South Korean Children

**DOI:** 10.1155/2019/7643850

**Published:** 2019-06-10

**Authors:** Hun Gu Choo, Sang Hoon Rah, Soo Han Kim

**Affiliations:** Department of Ophthalmology, Yonsei University Wonju College of Medicine, Wonju, Republic of Korea

## Abstract

**Purpose:**

To investigate the association between regional solar radiation and myopia occurrence in South Korean children.

**Materials and Methods:**

A population-based cross-sectional study using data of 1218 children aged 7–9 years from the Korea National Health and Nutritional Examination Survey was conducted from January 1, 2008, to December 31, 2012. Myopia prevalence and the mean spherical equivalent were estimated; myopia was defined as spherical equivalent refraction <−1.5 D. Data regarding solar radiation and sunshine duration were collected from 21 national monitoring stations in South Korea. Multiple logistic regression analyses and multiple linear regression analyses were used to evaluate the associations. However, the most important covariate, the time spent outdoors, was not measured and could not be used.

**Results:**

In the entire cohort of 1218 participants, solar radiation and sunshine duration were significantly associated with the mean spherical equivalent (*P*=0.001 and *P*=0.014, *B* = 0.088 and *B* = 0.069, respectively) and solar radiation was significantly associated with myopia prevalence (*P*=0.008). And, a negative but not statistically significant association between sunshine duration and myopia prevalence was observed (*P*=0.064, respectively).

**Conclusions:**

Solar radiation and sunshine duration are associated with the mean spherical equivalent and myopia prevalence in South Korean children.

## 1. Introduction

Myopia is a significant public health concern worldwide, especially in East Asian countries [1]. High myopia may lead to potentially blinding complications, such as retinal tears, myopic macular degeneration, and choroidal neovascularization in both adults and adolescents [2]. In the latter part of the 20th century, in highly urbanized East Asian regions, the prevalence of myopia increased dramatically and, in some highly educated populations, the prevalence of myopia currently exceeds 80% [3]. Thus, identification of factors that could increase the risk for myopia has been a focus of several studies. Family history of myopia and East Asian ethnicity have been proposed as possible risk factors [3]. In addition, atropine eye drops, which help correct peripheral hyperopia, decreased near work, and increased daylight exposure, have been proposed as protective factors that help prevent myopic progression [4]. Especially, sunlight exposure by outdoor activity is considered to be an important factor, which has been investigated by several studies [5–7]. In one study that involved 6-year-old Chinese children, the prevalence rate was 29.1% in those living in Singapore, whereas it was only 3.3% in those living in Sydney, Australia. The main reason for this disparity was the substantial difference in time spent outdoors between these two groups (13.8 h per week in Sydney vs. 3.0 h per week in Singapore) [8]. In addition, increased daylight exposure is thought to be an important protective factor against myopia, and several studies, including animal studies, have been recently conducted to explain the involved mechanism; furthermore, randomized clinical trials of outdoor interventions for myopia have also been conducted [9–11]. Several interventional studies have shown that outdoor activity prevents myopia progression [6, 12], and other studies have found that light intensity affects the degree of myopia [10, 11] and short wavelength is also considered as an important factor that affects the degree of myopia [13–16]. Conversely, one study reported that outdoor activity does not affect myopia [17].

However, to date, no study has evaluated the association of regional solar radiation and sunshine duration with myopia. Therefore, through the present study, we aimed to investigate the associations of regional solar radiation and sunshine duration with myopia prevalence and the mean spherical equivalent.

## 2. Materials and Methods

The Korea National Health and Nutrition Examination Survey (KNHANES) is an ongoing, nationwide, population-based, cross-sectional epidemiologic survey consisting of the following three parts: a health interview survey, health examination survey, and nutrition-related survey. A field survey team that included an ophthalmologist and nurse examiners for health assessments used a mobile examination unit and performed interviews and physical examinations [8]. Data of 1218 children aged 7–9 years were collected from January 1, 2008, to December 31, 2012. The presence of refraction without cycloplegia was assessed using an autorefractor (KR-8800®; Topcon, Tokyo, Japan) by ophthalmology residents or ophthalmologists. The refraction test was performed once per children, and step of measured refraction is 0.25 diopters (D). Refraction measurements were converted into spherical equivalents, which were calculated as the spherical value plus half of the astigmatism value. Myopia was defined as ≤−1.50 diopters (D) in either or both eyes. Solar radiation was defined as solar energy to be irradiated on a unit area, and sunshine duration was defined as time from sunrise to sunset. Data regarding solar radiation and sunshine duration gathered at 21 atmosphere-monitoring stations in South Korea from January 1, 1990, to December 31, 2009, were obtained from the Korean Ministry of Environment. The sum total of the mean annual solar radiation values for 20 years and the sum total of the mean annual sunshine duration values for 20 years recorded at the monitoring site located in the participants' residential areas were used as proxies of solar radiation and sunshine duration, respectively [18]. This study was approved by the Korea Centers for Disease Control and Prevention Institutional Review Board (IRB Number: 2008-04EXP-01-C, 2009-01CON-03-2C, 2010-02CON-21-C, 2011-02CON-06-C, 2012-01EXP-01-2C) and complied with the tenets of the Declaration of Helsinki. All participants provided written informed consent.

We considered traditional risk factors, including income level, regional factors, duration of near work activities, and outdoor activity, as covariates [19]. Furthermore, a previous study that used data from the KNHANES showed that education level was factors associated with myopia occurrence [20]. We recruited 7-year old to 9-year-old early elementary school students who spent limited time studying, rather than recruiting older, middle-school students, to control for education level and duration of near work activities.

All statistical analyses were performed using SPSS Complex Samples procedures (SPSS Statistics, version 21; IBM Inc., Armonk, NY, USA) according to the SPSS manual from the Korea Centers for Disease Control and Prevention. Pearson correlation analyses were used to evaluate the associations between solar radiation and sunshine duration with myopia prevalence and mean spherical equivalent. Multiple logistic regression analyses and multiple linear regression analyses were performed to control for the covariates (age, sex, and income level). *P* values < 0.05 were considered significant.

## 3. Results

**Figure 1:**
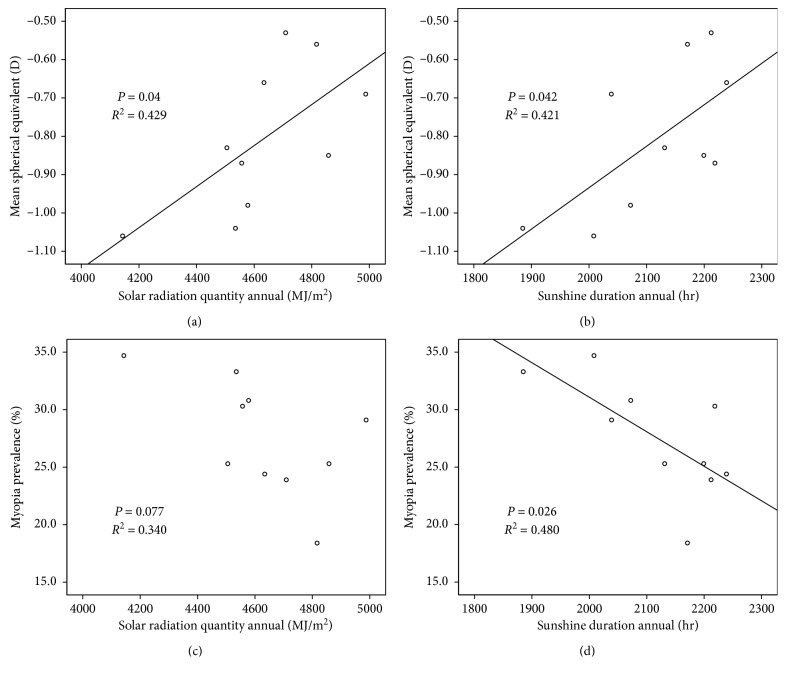
Scatter plot of solar radiation and sunshine duration with myopia degree and myopia prevalence.

The baseline characteristics of the study participants are shown in Table 1. Data of 1218 children were collected from the KNHANES. In the 1218 participants, solar radiation and sunshine duration were significantly associated with mean spherical equivalent (*P*=0.04 and *P*=0.042, respectively). In addition, negative but not statistically significant associations of solar radiation and sunshine duration with myopia prevalence were observed (*P*=0.04 and *P*=0.042, *R*^2^ = 0.429 and *R*^2^ = 0.421, respectively) and sunshine duration was significantly associated with myopic prevalence (*P*=0.026, *R*^2^ = 0.480, respectively); however, there was no statistically significant association between solar radiation and myopic prevalence (*P*=0.077, *R*^2^ = 0.340, respectively) (Figure 1).

**Figure 2:**
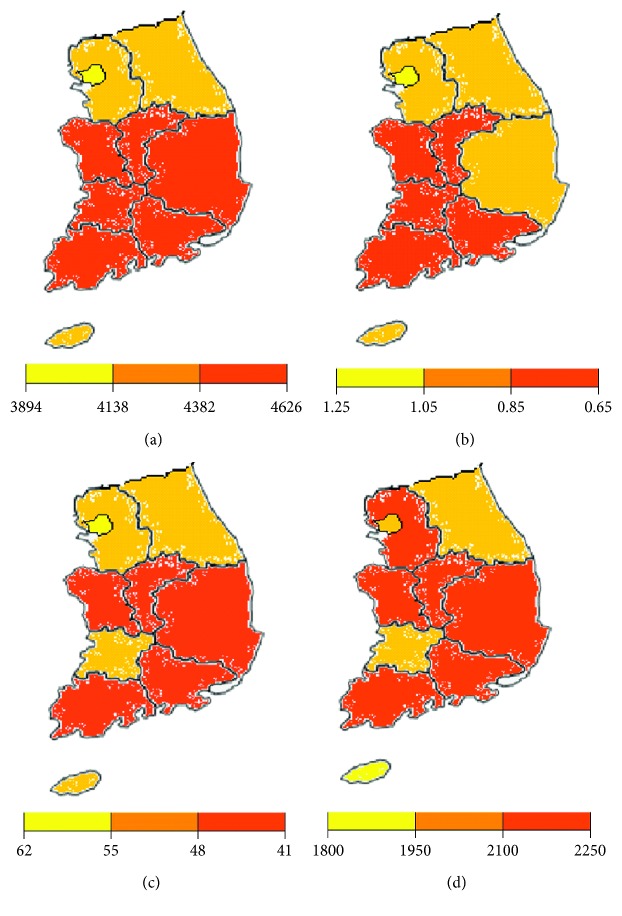
(a) Annual solar radiation, (b) mean spherical equivalent, (c) myopia prevalence, and (d) annual sunshine duration have similar a distribution pattern in South Korea.

This relationship can also be seen in maps showing regional solar radiation, sunshine duration, mean spherical equivalent, and myopia prevalence.  Figure 2 shows that there are similar distribution patterns in South Korea.

In the multiple linear regression analysis performed to control for socioeconomic factors (age, sex, and income level), solar radiation was significantly associated with the mean spherical equivalent (*P*=0.001, *B* = 0.088, respectively) and sunshine duration was significantly associated with the mean spherical equivalent (*P*=0.014, *B* = 0.069, respectively; Table 2). In the multiple logistic regression analysis performed to control for socioeconomic factors (age, sex, and income level), solar radiation was significantly associated with myopia prevalence (*P*=0.008, respectively) but sunshine duration showed a negative but not statistically significant association with myopia prevalence (*P*=0.064, respectively; Table 3).

## 4. Discussion

Exposure to sunlight has been recently postulated as a protective factor against myopia in children [10]. Further, children who spend time outdoors in regions where regional solar radiation is high and sunshine duration is long are expected to obtain better protection against myopia than those who spend the same amount of time outdoors in regions where regional solar radiation is low and sunshine duration is short. Hence, the regional solar radiation level and sunshine duration are expected to be related to myopia occurrence; however, to date, no study has examined the association between regional solar radiation or sunshine duration and myopia occurrence.

This population-based study demonstrated that solar radiation and sunshine duration were associated with the degree of myopia presented as the mean spherical equivalent. Increased solar radiation was associated with myopia prevalence, but sunshine duration showed a negative but not statistically significant association.

In this study, solar radiation had a stronger association with myopia prevalence than did sunshine duration, probably because solar radiation is based on both intensity and duration of light and light intensity is an important factor that provides protection against myopia [10, 11].

Multiple previous studies have reported an association between refractive error and sunlight exposure [3, 21, 22]. Furthermore, multiple animal studies have shown that high-intensity light prevents the onset and progression of myopia [10, 11].

Multiple theories explain the effect of sunlight on myopia. Recently, the retinal dopamine system has been proposed as a key mechanism [11]. High outdoor light intensity would result in greater depth of field and less image blur. Furthermore, release of dopamine from the retina is known to be stimulated by light; dopamine can act as an inhibitor of eye growth [3, 23], and it has been reported that dopamine release in mammals increases with increasing light intensity [24].

There are some limitations to this study. First, it was difficult to control for the entire set of covariates. In the KNHANES, no data are included on parental myopia and the time children spent engaged in outdoor activities. Thus, we could not consider the effect of parental myopia. Moreover, although we analyzed the data of 7-year-old to 9-year-old children who spent limited time studying to minimize the effect of near work and differences in lifestyle, this was not sufficient to control for the covariates. Second, this study had a cross-sectional design; thus, the results do not definitively indicate a cause-and-effect relationship between sunlight and myopia occurrence but rather only indicate an association. Third, we used 20 years of solar radiation data; however, it would have been better to use the solar radiation data from the last 10 years, as this would better reflect the effect of solar radiation on myopia in the children involved in this study, but unfortunately, we could not obtain those data. However, as the difference in regional sunshine is due to latitude and altitude in each region, it is not unreasonable to use the 20-year data if the geographical characteristics, including the latitude and altitude of each region, have not changed. Moreover, other studies examining solar radiation, such as solar power generation, are usually based on 20-year average data [25]. Fourth, this study did not use cycloplegic refraction data and noncycloplegic refraction may have caused measurement errors. In this case, biometrical data such as axial length could be used, but these could not be measured either. Thus, as in previous studies, we applied a wider myopia definition of −1.5 D [12].

Despite these limitations, we believe that this study is important because, to our knowledge, it is the first study to compare regional solar radiation and regional myopia prevalence. The present study indicated a negative association of solar radiation and sunshine duration with myopia degree, and this needs to be analyzed further by controlling the covariates better.

## Figures and Tables

**Table 1:** Baseline characteristics of the study participants.

Region	Total (*n* = 1218)			Annual solar radiation (MJ/m^2^)	Annual sunshine duration (h)
	Mean SE	Myopia prevalence (%) (defined as ≦−1.50 D)	Myopia prevalence (%) (defined as ≦−0.50 D)		
Seoul	−1.06 + 1.49	34.7	55.3	4143	2008
Gyeonggi-do	−0.87 + 1.39	30.3	50.6	4557	2219
Gangwon-do	−0.98 + 1.90	30.8	50	4578	2072
Chungcheongbuk-do	−0.53 + 1.11	23.9	42.6	4709	2212
Chungcheongnam-do	−0.56 + 1.09	18.4	46.1	4817	2171
Jeollabuk-do	−0.69 + 1.09	29.1	48.2	4987	2039
Jeollanam-do	−0.83 + 1.17	25.3	50.7	4505	2131
Gyeongsangbuk-do	−0.85 + 1.46	25.3	46.7	4858	2199
Gyeongsangnam-do	−0.66 + 1.62	24.4	46.8	4634	2239
Jeju-do	−1.04 + 1.74	33.3	43.3	4535	1885

SE, spherical equivalent.

**Table 2:** Multiple linear regression analysis to assess the associations of different variables with mean spherical equivalent.

Socioeconomic factor	Total (*n* = 1218)	
	*B*	*P* value
Annual solar radiation		
Age	−0.243	<0.001
Sex (female vs. male)	−0.016	0.557
Income level (high vs. low)	−0.061	0.027
Sunlight factor		
Solar radiation (MJ/m^2^)	0.088	0.001

Annual sunshine duration		
Age	−0.243	<0.001
Sex (female vs. male)	−0.017	0.550
Income level (high vs. low)	−0.059	0.033
Sunlight factor		
Sunshine duration (h)	0.069	0.014

**Table 3:** Multiple logistic regression analysis to assess the associations of different variables with myopia prevalence.

Socioeconomic factor	Total (*n* = 1218)	
	Odds ratio (95% CI)	*P* value
Annual solar radiation		
Age	2.065 (1.751–2.437)	<0.001
Sex (female vs. male)	0.986 (0.763–1.275)	0.917
Income level (high vs. low)	1.256 (0.960–1.644)	0.096
Sunlight factor		
Solar radiation (100 MJ/m^2^ increase)	0.933 (0.886–0.982)	0.008

Annual sunshine duration		
Age	2.060 (1.747–2.429)	<0.001
Sex (female vs. male)	0.985 (0.763–1273)	0.910
Income level (high vs low)	1.245 (0.952–1.629)	0.110
Sunlight factor		
Sunshine duration (100 h increase)	0.887 (0.782–1.007)	0.064

CI, confidence interval.

## Data Availability

The National Health and Nutrition Examination Survey is available on the following websites at https://knhanes.cdc.go.kr/knhanes/sub03/sub03_02_02.do. Solar radiation and sunshine duration data are available on the Korean Ministry of Environment websites at http://www.weather.go.kr/weather/climate/solar_energy01.jsp?type=1&element=9&x=19&y=1&year=2010.
